# Post-traumatic Trigeminal Neuropathic Pain: Factors Affecting Surgical Treatment Outcomes

**DOI:** 10.3389/froh.2022.904785

**Published:** 2022-07-07

**Authors:** Timothy W. Neal, John R. Zuniga

**Affiliations:** Division of Oral and Maxillofacial Surgery, Department of Surgery, University of Texas Southwestern Medical Center, Dallas, TX, United States

**Keywords:** neuropathic pain, chronic pain, trigeminal nerve, microsurgery, surgical outcomes

## Abstract

Post-traumatic trigeminal neuropathic pain (PTTNp) is a painful condition that may result from injury to the sensory division of the trigeminal nerve. Treatment of this condition is challenging and consensus on treatment to resolve neuropathic pain has yet to be standardized. Equally as challenging is the identification of surgical outcome variables to guide surgical treatment of PTTNp. This is partly due to the variability in pain characteristics, severity of nerve injury, location, and duration from injury to surgery. In those with neuropathic pain prior to microsurgical intervention, the incidence of neuropathic pain after microsurgical intervention is 67%. It is unclear why nerve repair surgery is effective in resolving or decreasing neuropathic pain in some patients, whereas it has no effect on pain relief in others. Psychological, medical, and age-related factors have been identified as risk factors for developing chronic post-surgical pain due to post-traumatic neuropathic pain. Two factors: injury to surgery time and preoperative visual analog scale score have recently been identified as variables that influence surgical outcomes in the treatment of PTTNp.

## Introduction

Iatrogenic injury to the trigeminal nerve may occur following elective dental or oral and maxillofacial surgery procedures. The branches of the trigeminal nerve usually affected are the inferior alveolar nerve (IAN) and the lingual nerve (LN) [1, 2]. These injuries are most commonly caused by third molar extraction, followed by orthognathic surgery, mandibular trauma, dental implant placement, local anesthesia injection, endodontic therapy, and pathology resection [2]. The reported incidence of IAN or LN injury following these procedures is wide ranging at 0.6 to 90% [3–5]. When the IAN is injured, the most common outcome is loss of general sensation to the mouth, teeth, lip, and chin. Lingual nerve injuries may result in loss of general sensation and special sensation to the anterior two-thirds of the tongue as well as loss of general sensation to the lingual mucosa and floor of mouth. Depending on the mechanism and severity of injury, most injured IANs and LNs recover spontaneously [6, 7]. When injury is severe enough to require surgical intervention, the success rate of microsurgery is high (90%) depending on factors such as time from injury, degree of nerve injury, location of injury, and age of the patient [8, 9].

Following injury or insult to the trigeminal nerve, some patients experience post-traumatic trigeminal neuropathic pain (PTTNp). PTTNp is a painful condition characterized by allodynia, hyperpathia, and/or hyperalgesia. Allodynia is a painful response to a normally nonpainful stimuli, hyperpathia is a complex painful response to repetitive nonpainful stimulus, and hyperalgesia is a lowered pain threshold to a painful stimulus. The cause of PTTNp is still unknown but animal studies have implicated various biological processes such as inflammation, enhanced neuropeptide-mediated pain signal transmission, endothelial receptor activity, and glial cell dysfunction causing trigeminal hyperexcitability [10]. PTTNp may be diagnosed clinically by clinical neurosensory testing as described by Zuniga and Essick [11]. The reported incidence of PTTNp following injury to the IAN or LN is 0.45–70% [12, 13]. PTTNp is a devastating condition that has substantial impact on quality of life. It has been shown longitudinally that patients with PTTNp report moderate to severe pain, and that this pain correlates with lower quality of life and depression [14, 15]. The treatment of PTTNp is challenging, and consensus on treatment to resolve neuropathic pain has yet to be standardized. Non-surgical treatment of neuropathic pain including pharmacological, non-pharmacologic, neuromodulatory, and behavioral based care has not provided pain relief in most patients suffering from neuropathic pain and many of these patients experience adverse effects related to treatment medications [16, 17]. Surgical interventions including neuroma resection, neurolysis, and neurorrhaphy with or without autograft or allograft have had mixed outcomes and have provided only chance reduction or elimination of neuropathic pain. Identification of surgical outcome variables to guide treatment is challenging, in part due to the variability in pain characteristics, severity of nerve injury, location, and duration from injury to surgery. Previous reports investigating surgical outcomes have not identified phenotypic markers that predict relief or reduction of pain following surgical treatment of PTTNp. Two factors: injury to surgery time and preoperative visual analog scale score, have recently been identified as variables that influence surgical outcome in the treatment of PTTNp.

## Development of PTTNp

A number of risk factors for the development of PTTNp following various oral and maxillofacial surgery procedures have been identified in the literature. Marchiori et al. found that gender, older age, and depression were risk factors for developing neuropathic pain after orthognathic surgery [11]. In a study of patients that underwent microsurgery of the IAN, Bagheri et al. found that patients with pain prior to surgery were more likely to be female, had a greater duration from injury to repair, and were more likely to have nerve compression injuries [8]. In a study of patients that presented for microsurgical repair of the trigeminal nerve, Zuniga et al. found that patients who presented with pain were older and had IAN injuries of class 3 Sunderland variation when compared to patients with no pain [18].

Patients that experience PTTNp often report moderate to severe pain levels and the symptoms typically persist. In a study by Van der Cruyssen et al. of patients with PTTN, 86.2% of patients still complained of symptoms after 2 years. The greatest improvement in symptoms occurred during the first 20 weeks after injury with little improvement seen after 60 weeks and lingual nerve injuries had the best long-term outcomes when compared to IAN injuries [14, 15].

## Preoperative Pain Intensity

Patients that present for microsurgical treatment of PTTNp report variable pain levels typically in the moderate to severe range. As a predictor of surgical outcome, it has been shown that preoperative neuropathic pain predicts postoperative neuropathic pain with a positive predictive value of 67% [18]. The association of preoperative pain intensity and surgical outcome was studied previously by Zuniga et al. Twenty-eight patients who underwent trigeminal nerve repair of the inferior alveolar nerve and lingual nerve with documented preoperative neuropathic pain were divided into three groups: complete recurrence (*n* = 10), incomplete recurrence (*n* = 11), and no recurrence (*n* = 7). Among the 3 groups there was no statistical difference in preoperative pain intensity (*p* = 0.16). However, there were statistical differences at 3 months postoperative (*p* = 0.007), 6 months (*p* < 0.001), and 12 months (*p* < 0.001). The average preoperative pain scores of patients with no recurrence of PTTNp post-surgery was 5.57, incomplete recurrence was 7.5, and complete recurrence was 6.5. Following microsurgery if there was recurrence of PTTNp it occurred in the first 6 postoperative months. A limitation of this study was that it was underpowered [19].

**Figure 1:**
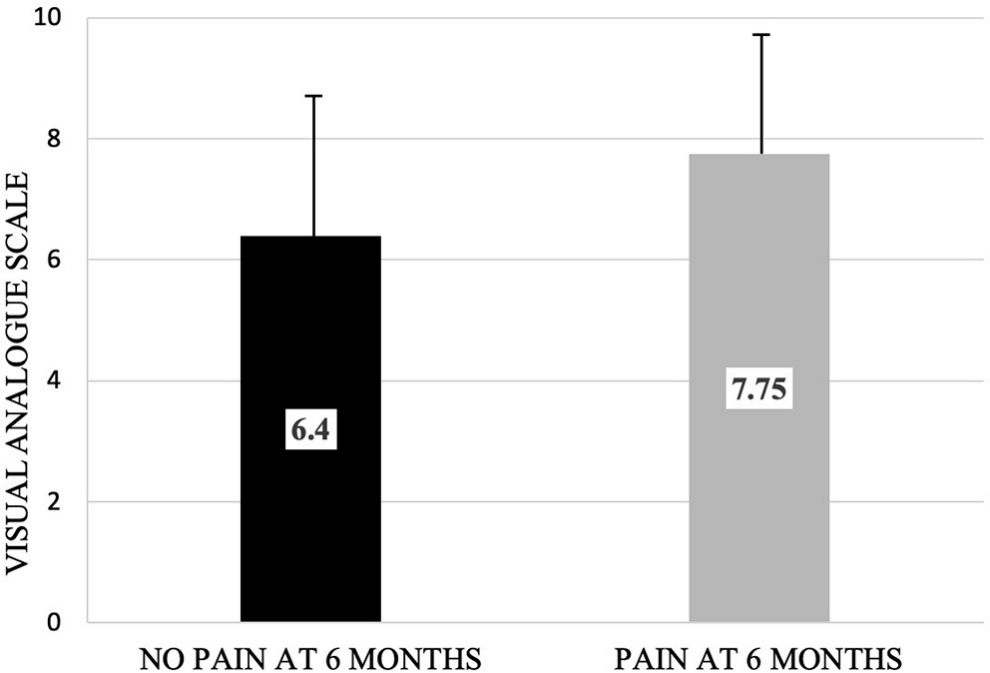
Preoperative pain level of cohort with no neuropathic pain at 6 months post-surgery visit and cohort with neuropathic pain at 6 months post-surgery visit as measured by visual analog scale. Bars depict upper limit of standard deviation (No pain: 2.68; Pain: 1.95). There was a statistically significant difference in preoperative pain level between the two groups (*p* = 0.0412).

Recently, the current authors investigated the effect of preoperative pain scores on surgical outcomes in patients with PTTNp. The major improvement of this study was the increase in power. The population was composed of patients that underwent elective trigeminal nerve repairs of the IAN or LN from 2006 to 2021. Patients included in the study had: 1. Neurosensory complaints in the IAN or LN distribution following third molar extraction, dental implant placement, endodontic treatment, orthognathic surgery, or mandibular trauma; 2. Clinical neurosensory testing (NST) and/or Magnetic Resonance Neurography (MRN) findings of a Sunderland Class II to V injury; 3. Clinical neurosensory test-based diagnosis of neuropathic pain that resolved with local anesthetic blocks to the injured nerve preoperatively; 4. pain intensity measured with VAS scores were available preoperatively and at 6 months postoperatively; and 5. any age, gender, or race. All patients included received no post-surgical medical, behavioral, neuromodulatory or neurosurgical management. Patients were excluded if they had any of the following: 1. acute infection at the time of surgery; 2. history of radiation therapy to the head and neck; 3. current or previous malignancy of the head and neck; 4. medication-induced osteonecrosis of the jaw; 5. demonstrated persistent PTTNp after peripheral trigeminal nerve blocks or in response to control nerve blocks; 6. did not have postsurgical neurosensory and neuropathic testing. Fifty-three patients with preoperative PTTNp of either the LN or IAN who underwent microsurgical treatment were divided into two groups: PTTNp present at 6 months and PTTNp not present at 6 months. The average pain level as measured by Visual Analog Scale (VAS) in the group with no pain at 6 months was 6.4, while the group with pain at 6 months had an average score of 7.75 (Figure 1). There was a statistically significant difference in the preoperative pain intensity between the two groups (*p* = 0.0412). As well, in the group with pain at 6 months there were more IAN injuries, and a longer duration from nerve injury to microsurgical repair.

## Injury to Surgery Time

Time is a known factor that influences outcomes of nerve surgery. In cases of severe nerve injury that require repair, functional sensory recovery is higher in patients that undergo microsurgical repair within the first 3 months following injury [20, 21]. Intuitively it makes sense that this principle of early repair would apply to patients who present with PTTNp. It has been shown that patients who present with PTTNp typically have longer injury to surgery duration compared to those that have no pain [8]. In an ambispective study by Zuniga et al. comparing a cohort with PTTNp prior to microsurgical repair (*n* = 17), to a cohort with no pain (*n* = 48), there was no significant difference in injury to surgery duration between the two groups. The study noted, however, that injury to surgery time was nearly significant and may be a significant variable in a larger cohort of patients [18].

**Figure 2:**
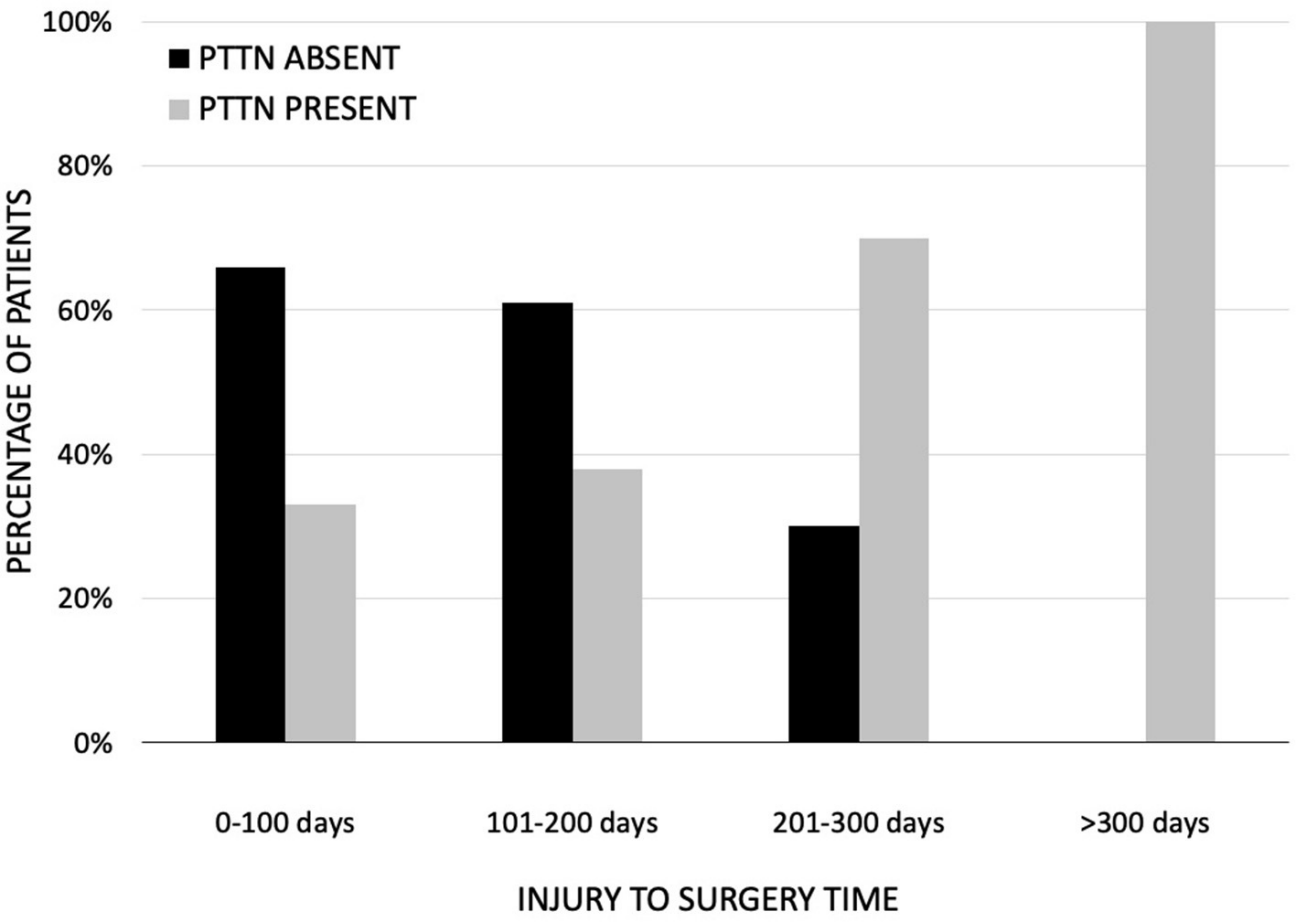
Absence and presence of neuropathic pain at 6 months postsurgical follow up. When the time to surgery was 200 days or less, the percentage of patients with neuropathic pain before surgery with no neuropathic pain at the 6 month follow up was greater than 60%.

The current authors recently investigated the effect of time on surgical outcomes in patients with preoperative PTTNp. The population was composed of patients that underwent elective trigeminal nerve repairs of the IAN or LN from 2006 to 2021. The inclusion and exclusion criteria were the same as previously described in this article. Sixty-three patients with preoperative PTTNp who underwent microsurgical repair were divided into four cohorts based on time from injury to surgery: Cohort 1 (0–100 days); Cohort 2 (101–200 days); Cohort 3 (201–300 days); and Cohort 4 (>300 days). The primary outcome was the presence or absence of PTTNp at the 6-month postoperative visit. There was statistical difference in the primary outcome between the cohorts (p = 0.0002). It was found that when the time from injury to surgery was 200 days or less, the percentage of patients with PTTNp before surgery with no neuropathic pain at the 6 month follow up was greater than 60%. When the injury to surgery time was greater than 200 days, just 12% of patients had no PTTNp at 6 months follow up (Figure 2).

## Discussion

In the treatment of nerve injuries, time is an important factor. Recent findings from our group show that operative delay has a negative impact on the surgical treatment of PTTNp. As well, patients with recurrence of PTTNp at 6 months often have more intense pain prior to surgery. It was also found that in patients with more intense pain prior to surgery, there was a longer injury to surgery duration. This may imply that if PTTNp persists, pain intensity may increase over time. While these findings are promising, there remains the fact that some patients with PTTNp recover spontaneously or by non-surgical treatment following nerve injury [12]. Before consensus on surgical treatment and timing is reached, further studies are needed to evaluate the effect of time from injury to repair on PTTNp within the 200-day window. As well, further studies evaluating pain intensity in patients with PTTNp as it relates to time are needed. With no previous phenotypic markers that predict relief or reduction of PTTNp following microsurgery, these factors provide points of future study that may improve the predictability of PTTNp microsurgical treatment.

## Data Availability Statement

The original contributions presented in the study are included in the article/supplementary material, further inquiries can be directed to the corresponding author.

## Ethics Statement

Ethical review and approval was not required for this study in accordance with the local legislation and institutional requirements.

## Author Contributions

TN and JZ wrote, critically read, and edited the manuscript. Both authors contributed to the article and approved the submitted version.

## Conflict of Interest

JZ is a paid consultant for AxoGen Inc., but this submission was not supported by or reviewed by AxoGen Inc. The remaining author declares that the research was conducted in the absence of any commercial or financial relationships that could be construed as a potential conflict of interest.

## Publisher's Note

All claims expressed in this article are solely those of the authors and do not necessarily represent those of their affiliated organizations, or those of the publisher, the editors and the reviewers. Any product that may be evaluated in this article, or claim that may be made by its manufacturer, is not guaranteed or endorsed by the publisher.
